# Trichromatic perception of flower colour improves resource detection among New World monkeys

**DOI:** 10.1038/s41598-018-28997-4

**Published:** 2018-07-18

**Authors:** J. D. Hogan, L. M. Fedigan, C. Hiramatsu, S. Kawamura, A. D. Melin

**Affiliations:** 1 University of Calgary, Calgary, Canada; 2 Kyushu University, Fukuoka, Japan; 3 University of Tokyo, Kashiwa, Chiba, Japan

## Abstract

Many plants use colour to attract pollinators, which often possess colour vision systems well-suited for detecting flowers. Yet, to isolate the role of colour is difficult, as flowers also produce other cues. The study of florivory by Neotropical primates possessing polymorphic colour vision provides an opportunity to investigate the importance of colour directly. Here we determine whether differences in colour vision within a mixed population of wild dichromatic and trichromatic white-faced capuchins (*Cebus capucinus imitator*) affect flower foraging behaviours. We collected reflectance data for flower foods and modelled their chromatic properties to capuchin colour vision phenotypes. We collected behavioural data over 22 months spanning four years, determined the colour vision phenotype of each monkey based on amino acid variation of the L/M opsin gene from fecal DNA, and compared foraging behaviours of dichromats and trichromats. Most flowers were more conspicuous to trichromats, and trichromats foraged in small flower patches significantly more often. These data demonstrate a difference in wild primate foraging patterns based on colour vision differences, supporting the hypothesis that trichromacy enhances detection of small, ephemeral resources. This advantage, which may also extend to other foods, likely contributes to the maintenance of colour vision polymorphism in Neotropical monkeys.

## Introduction

Many plants use chromatic signals to attract pollinators, and many pollinators, including birds and butterflies, have colour vision abilities that seem well-suited for this task^1–4^. Yet, flowers also produce odour and luminance cues to attract animals, and the specific role of colour vision in locating flowers can be hard to assess in the wild^1,2^. New World monkeys provide a unique opportunity to investigate the role of colour in attracting animal visitation to flowers. Neotropical primate species have variable colour vision due to allelic polymorphism of the long-to-mid wavelength (*OPN1LW*) opsin gene, and live in social groups consisting of both trichromats and dichromats. Variation in this single-copy, X-linked gene results in all males and homozygous females having dichromatic vision, while heterozygous females possess trichromacy^5^. Such polymorphism provides an ideal system to investigate the adaptive significance of colour for the detection of food resources, and a large and diverse body of literature has examined this topic^6–18^. An emerging consensus is that: (1) discrete advantages are possessed by trichromats for feeding on reddish ripe fruits and by dichromats for cryptic resources, such as surface-dwelling insects; and (2) foraging ecology has likely influenced balancing selection acting on the *OPN1LW* opsin gene, maintaining colour vision polymorphism through negative frequency-dependent selection, mutual benefit of association, or niche divergence^19–21^. Furthermore, links between primate evolution and angiosperm radiation have long been hypothesized^22^, and several Neotropical primate species, including white-faced capuchins (*Cebus capucinus imitator*), may play important roles as pollinators^23–29^.

Improved detection of reddish-to-yellowish food items such as flowers, ripe fruits and young leaves is believed to have contributed to selective pressure shaping colour vision in primate evolution^13,20^. Recent research has demonstrated that, in specific contexts, trichromats feed more quickly on reddish fruits, feed for longer periods on ripe fruit, and have higher body mass^13,30^. However, it has been repeatedly predicted that the largest advantage of trichromacy lies in the detection of small, novel, ephemeral resources that are not contestable or memorable, a hypothesis that remains untested^13,16^. Due to the highly variable lighting conditions in a forest, flower chroma (hue and saturation) may be among the most reliable cues for long distance detection of floral resources^14^. However, cognitive and social dynamics likely obscure trichromatic advantage for large food patches, because these resources are memorable (and therefore located via spatial cognition) and contestable (therefore higher-ranking individuals may locate resources through following and displacing conspecifics)^14,16,31^. Food detection advantages resulting from trichromacy may therefore only be observable for foods produced in small, unmemorable patches that can be consumed quickly^31^.

In the present study we investigate whether differences in colour vision affect primate-flower interactions. If colour is important for detecting floral resources, then dichromatic and trichromatic primates should have different foraging efficiencies for flowers. We predict that the flowers consumed by Costa Rican white-faced capuchins are more detectable to trichromats than to dichromats against a leafy background, and that trichromats will dedicate a higher proportion of their daily activity budget to florivory than will dichromats. We also test whether flowers in small, monopolizable patches are consumed more frequently by trichromats.

## Results

### Colour vision modelling results

**Figure 1:**
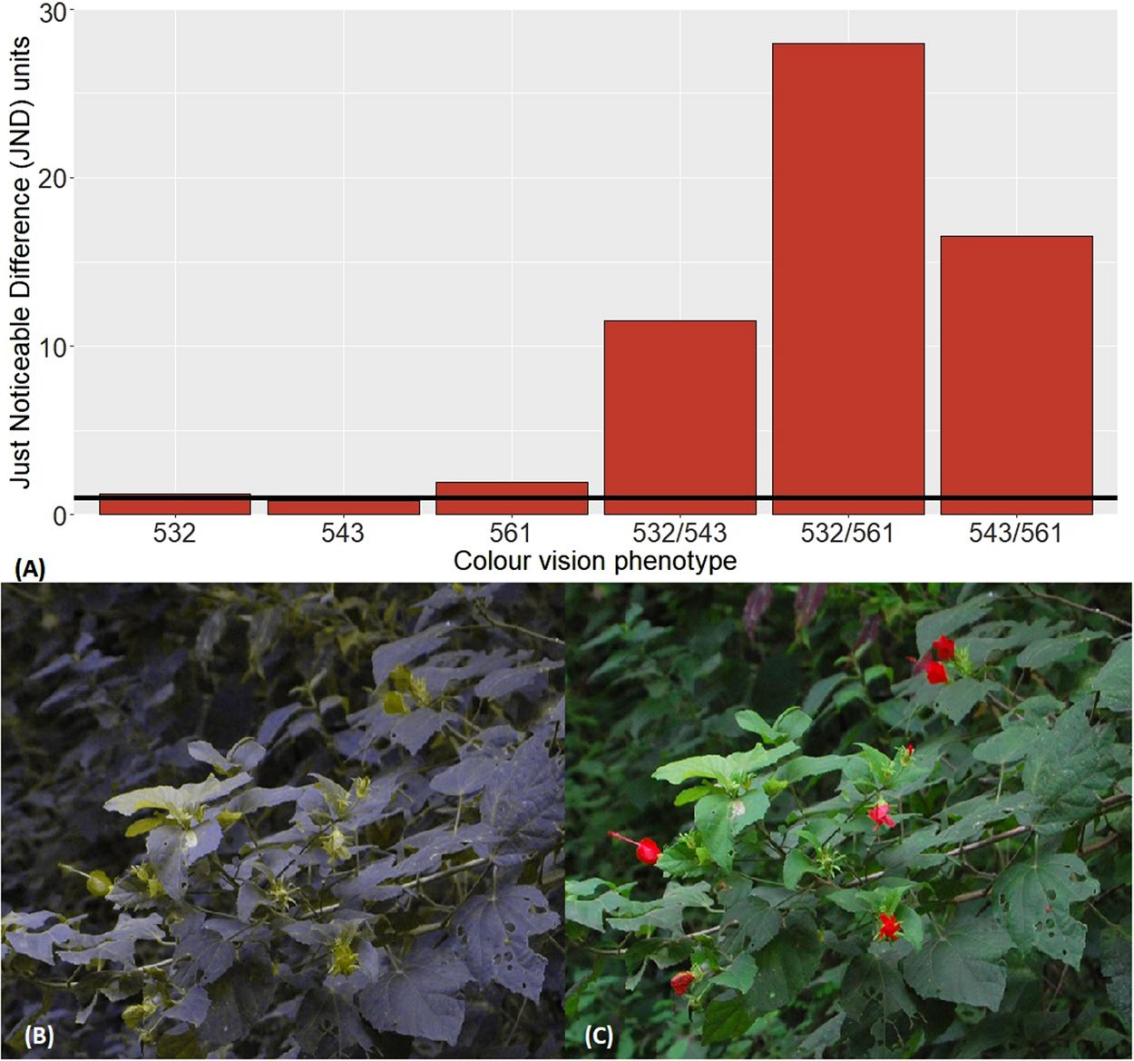
An example of the impact of colour vision phenotype on ability to discriminate flowers from leaves. We present the (**A**) chromatic Just Noticeable Difference (JND) scores for each capuchin colour vision phenotype for flowers of *Malvaviscus arboreus* relative to background leaves. JND values for all dichromat phenotypes are near 1 JND (the point at which an object is distinguishable from its background under ideal conditions; demarcated by the black horizontal line in this figure), while it is far more conspicuous from a leafy background to trichromats (JND values >11). Using a colour vision simulation software^52^, we further show that the flowers of this species appear more similar in colour to leaves for (**B**) dichromat individuals (λmax of ML-pigment: 532) than for (**C**) trichromat individuals (λmax of ML-pigments: 532/561).

Just Noticeable Difference modelling indicates that all flower parts of all tree species should be visible (i.e.; >1 JND) relative to a green leaf background for all three trichromatic phenotypes. Conversely, 3/21 flower parts did not differ from green leaves by >1 JND for dichromats, and another 5 were only marginally detectable (i.e.; <2 JND; Supplemental Data S1). Mean JND values were higher for trichromats (5.2 JND) than dichromats (2.8 JND), and all 3 trichromat phenotypes had at least a 1 JND advantage for most flower species and analyzed parts (Supplemental Data S1 and Table 1). Trichromats of the 532/561 phenotype had detection advantages for 11 of 14 species and 15 of 21 flower parts relative to dichromat phenotypes. Despite the reduced range of spectral sensitivity, trichromats of phenotype 532/543 and 543/561 also maintain detection advantages compared to dichromats for many flower parts (Supplemental Data S1 and Table 1). Trichromats of all three phenotypes had a particularly strong predicted ability to perceive red flowers such as *Malvaviscus arboreus* (11–28 JND), a species that was borderline for dichromats (0.75–2.16 JND; Fig. 1).

All Support Vector Machine (SVM) models using all three trichromatic phenotypes were able to correctly categorize a majority of flowers, whereas most flowers were incorrectly categorized in dichromatic models for the three dichromatic phenotypes (Supplemental Data S2). The majority of flower parts (12/21) had luminance values that overlapped with sampled leaves. Adding lightness to the SVM models increased the success of all dichromats and slightly decreased trichromatic success (Supplemental Data S2). The white-and-green flowers of *Luehea speciosa* were not particularly visible to any phenotype, and were not correctly categorized in most instances of SVM modelling.

### Frequency of flower foraging by phenotype

**Figure 2:**
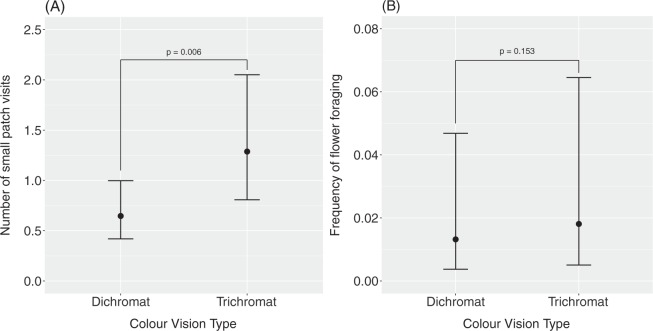
The (**A**) least square means estimate of the number of small patch visits to flowers per individual and (**B**) least square means estimate of the frequency of flower foraging observed for dichromatic and trichromatic white-faced capuchins. Trichromats were significantly more likely to be observed foraging in a small, conspicuous flower patch, while there was no significant difference in the overall flower foraging frequency between phenotypes.

We observed 423 instances of capuchins foraging for flowers from 15 plant species over the duration of the study periods. We recorded a total of 109 flower patch visits (FLPVs) to 8 species of small-patch flower species for 61 individual monkeys (33 dichromats and 28 trichromats). As predicted, trichromats were observed in these small flower patches significantly more than dichromats relative to their respective abundances in the study groups: trichromats were nearly twice as likely to be encountered in a small flower patch than were dichromats (Estimate = 0.6882, SE = 0.2449, z = 2.754, p = 0.006; Fig. 2A). Dichromats and trichromats spent similar amounts of time (as measured by the proportion of scan samples per sampling cycle dedicated to florivory) foraging on flower resources. The number of flower foraging scans by each phenotype did not significantly differ (Estimate = 0.3153, SE = 0.2208, z = 1.428, p = 0.153; Fig. 2B). While florivory accounted for only 0.4% of the annual activity budget as measured by scan sampling, seasonal flower use could be much higher (see Hogan *et al*.^24^ for a detailed account of the florivory patterns in this study population).

## Discussion

Flowers consumed by capuchins were often found in small patches and consumed quickly and JND modelling suggests that many small patch species produced flowers for which trichromats have large detection advantages. That trichromats forage in such patches significantly more often than dichromats do provides behavioural evidence in support of this prediction and highlights the importance of colour as an attractant to floral resources. From afar, many cues (e.g., touch, olfaction^15,32^) that dichromats may use at close range to compensate for their reduced chromatic discrimination ability are ineffective. The L/M opsin alleles in this population of capuchin monkeys exhibit characteristics of balancing selection^19^, and previous studies have revealed other conditions for which dichromatic capuchins have foraging advantages^7,33^. However, our research is one of the first studies to find a clear trichromatic detection advantage, and indicates that foraging for small resources that are chromatically conspicuous in the long-wavelength range contributes to the maintenance of opsin variation and polymorphic trichromacy in Neotropical primates^19^. Many primates with polymorphic trichromacy are frugivorous, and we anticipate that trichromat detection advantages leading to differential foraging may also be found for small fruit patches, a topic worth further investigation^16^.

It is important to note that not all flowers are predicted to be more detectable by trichromats. The most frequently consumed flower species (*Luehea speciosa*, 75% of all flower foraging activity) produces white flowers with green bracts. While JND modelling predicted a small advantage for trichromats detecting this species, SVM modelling did not predict a trichromat advantage, and the overall time spent foraging for this species did not differ between dichromats and trichromats. However, *Luehea speciosa*, as well as other important flower species such as *Diphysa americana*, produce large crops of inflorescences in emergent canopies, and social dynamics and spatial memory are likely to be more important determinants of foraging activity than differential colour vision abilities^16,17^.

This study provides some of the first direct evidence of differential resource use between colour vision phenotypes in a wild primate population, and underscores the importance of investigating niche differences at a very fine scale^7^. While flowers are not a principal diet item, they are of high seasonal importance in the early dry season, during which 25% of the foraging time budget can be dedicated to florivory^24^. Flower use, and by extension other conspicuous small patch food items, especially ripe fruit^14^, may be an important factor favoring the evolution and maintenance of trichromatic colour vision. Care must be taken, however, not to conflate trichromatic advantage at a particular task (in this case flower foraging) with heterozygote advantage as a maintenance mechanism for polymorphic colour vision. Rather than a specific phenotype having a net fitness advantage in the wild, colour vision polymorphism may persist by improving the fitness of all individuals in a mixed group of dichromats and trichromats, either through niche partitioning (with trichromats and dichromats occupying slightly different niches to ease competitive pressures) or via mutual benefit of association (with trichromats and dichromats each offering detection abilities for different sharable food patches or predators, improving the fitness of all group-members^19^). Group living has been long considered to be an evolutionary trade-off between reducing predation risk at the expense of increasing feeding competition^34^. Polymorphic colour vision potentially aids with both facets of group living: trichromats and dichromats can rely on different foods during periods of low food abundance while also specializing in the detection of different predators^35,36^.

## Materials and Methods

### Study species

White-faced capuchins primarily eat ripe fruit and invertebrates, but they are known to consume flowers in high abundances seasonally (i.e., up to 25% of the monthly plant diet^24^). Capuchins have polymorphic colour vision, and possess three long-to-mid wavelength sensitive (L/M) cone types with peak spectral sensitivities at 532 nm, 543 nm, and 561 nm^37^. All males and homozygous females are dichromats, whereas heterozygotic females exhibit trichromacy. All behavioural data were collected in accordance with protocols approved by the University of Calgary’s Life and Environmental Care Committee (LESACC).

### Study site and animals

We obtained behavioural and spectral data from Sector Santa Rosa (SSR), Área de Conservación Guanacaste (ÁCG), Costa Rica. This study was conducted with permission from the Government of Costa Rica, and Área de Conservación Guanacaste administration. SSR is primarily tropical dry forest characterized by strong variation in precipitation. Although there is seasonal variation in overall flower abundance in SSR, flowers are relatively abundant throughout the year, and many plant species produce flowers during the dry season when other capuchin foods are less abundant^24^.

This study combines two field seasons: 2007–2008 and 2013–2014. In 2007–2008 we studied four habituated capuchin groups (CP, EX, GN, LV) over 13 months between January 2007 and August 2008. Between May 2013 and March 2014, we followed three groups (AD, LV, RM) for 8 months. We followed each group for 2–4 consecutive full days (typically 12 hours/day) for each “cycle”, with one cycle per month in 2007–2008 (n = 13 cycles) and two per month in 2013–2014 (n = 13 cycles). Study groups are well habituated and individual monkeys are identifiable. Most individuals had been previously genotyped for the L/M opsin from fecal DNA, and we genotyped new individuals following the same protocols^14,37^. Individuals of each of the six possible colour vision phenotypes are represented in our study (Table 1). Researchers and assistants collecting behavioural data were not informed of any monkey’s phenotype to minimize observer bias.

**Table 1:** Distribution of colour vision phenotypes in the study population of white-faced capuchins (N = 132).

Phenotype	Number of individuals
Dichromat 532	11 (0 ♀/11 ♂)
Dichromat 543	17 (2 ♀/15 ♂)
Dichromat 561	45 (19 ♀/26 ♂)
Trichromat 532/543	6 ♀
Trichromat 532/561	24 ♀
Trichromat 543/561	7 ♀

### Colour measurement and modelling

We collected reflectance spectra from flowers and leaves using a USB 4000 portable spectrometer and LS-1 light source (2007–2008) and a Jaz EL-200 spectrometer with a PX pulsed xenon light source (2013–2014), from Ocean Optics Ltd. Both spectrometers read wavelengths between 200–850 nm, although we use only readings relevant to the capuchin visual system (400–700 nm^38^) in this study. Spectrometers were calibrated using a WS-1 reflectance standard at the start of each measurement session, and every 15 minutes during use to prevent drift over time. Measurements were taken using the reflectance function of Ocean Optics SpectraSuite software (64-bit version 1.6.0.11). To account for baseline drift via dark correction, we standardized our data by setting the lowest reading within the 400–700 nm range to 0 nm, and adjusting all others accordingly, using Microsoft Access 2010. Five separate samples were analyzed for each flower part whenever possible. If different flower parts were distinctly different colours to the trichromatic researcher’s eyes (e.g., *Centrosema macrocarpum* has white/cream coloured petals with a dark purple center), each colour patch was treated as a separate sample, as were the different flower maturity stages consumed by capuchins. In total we analyzed 21 flower parts from 14 flower food species (Supplemental Data S1). To simulate a heterogeneous leafy background that would best approximate SSR’s forest, we analyzed the chromatic properties of the upper and lower leaf surfaces from these 14 species plus the leaves from an additional 14 tree species common in the SSR forest.

### Just noticeable difference (JND) modelling

Predicting the conspicuity of objects to a non-human primate using subjective human colour categories is problematic, a shortcoming that can be addressed by modelling the response of specific cone sensitivities for each species or phenotype^37–40^. The maximum spectral sensitivities (λ_max_) of capuchin opsin pigments have previously been determined via measuring the absorbance spectra of pigments reconstituted *in vitro*^37^. Using these values, we estimated the absorbance spectra of each of the three visual pigments using the modified version of Lamb’s equation^41^ by giving the λ_max_ value of each visual pigment. To quantify how visible a flower was to each colour vision phenotype, we used Just Noticeable Difference (JND) modelling^37^ (Supplemental Data S1). The minimum chromatic distance at which two objects can be differentiated as discernibly different colours is defined as 1 JND^7,42^, and objects with increasingly higher JND scores are presumed to be visible under less ideal situations. We determined the JND scores of each flower target against the mean leaf background value for each capuchin colour vision phenotype. If a flower-leaf comparison scored >1 JND for a phenotype it was considered detectable, and scores at least 1 JND higher for one phenotype relative to another was considered advantageous.

To independently assess our JND modelling results we also analyzed our chromaticity data using “Support Vector Machine” (SVM) modelling (Supplemental Data S2), which uses a machine learning, leave-one-out approach to predict whether a given capuchin colour vision phenotype should be able to distinguish a flower part from a heterogeneous leafy background using the colour vision pathways available to that phenotype^14^ (Supplemental Data S2). In this analysis, each flower is plotted and assessed relative to all leaves separately.

### Flower foraging differences between phenotypes

We conducted instantaneous scan sampling to determine if time devoted to flower resource use differed among colour vision phenotypes^43^. Every half hour, investigators located as many monkeys as possible in 10 minutes and recorded their identity and behavioural state^43,44^. If a monkey was foraging, we recorded the type of food item whenever possible. We collected scan data on large juveniles, and adults/subadults.

We also collected data using a form of all-occurrences sampling technique we refer to as “flower patch visit” (FLPV) sampling, which is designed to capture rare, fleeting behaviours^14,24,44,45^. We scored any foraging by a capuchin group to a flower patch as one FLPV. When an FLPV was noted, observers recorded the flower species being consumed and the individual monkeys involved.

Using R software (64-bit version 3.3.0) and the lme4 statistical package^46^ (v. 1.1–11) we used a generalized linear mixed model (GLMM) to determine whether flower consumption frequency (the number of scan samples per study cycle in which flower foraging was recorded) was influenced by colour vision ability (trichromat or dichromat). Since this analysis uses count data with a high number of zeroes, we used a negative binomial distribution^47^. We included all adults, subadults and large juveniles present for the majority of a field year (i.e., at least 7 cycles of data collected in a study period of 13 cycles, n = 83 individuals). All individuals and study cycles were considered repeated measures, and the total sampling effort per individual was controlled by including an offset in the model, using the log of the total scans per individual per cycle. We used a minimum significance threshold of p < 0.05 for all analyses. We did not control for the phyletic relatedness of flower species in our models. However, the 14 species consumed during our study period were not closely related and were from 10 different families. Additionally, flower colouration varies considerably among closely related plant species as well as intraspecifically^48–50^; therefore, we do not believe this to be a major factor influencing our conclusions.

To examine whether trichromats were better able to locate small flower patches, we used a GLMM with negative binomial distribution to compare the number of times dichromats and trichromats were observed in FLPVs. For this analysis, to prevent the effects of contest competition obscuring detection-based foraging advantages, only visits to plant species producing small flower crops were included. A plant species was defined as a small patch if it only produced a small number of flowers that provided a limited and quickly-depleted patch. A total of 11 species previously identified as capuchin flower foods met these criteria: *Bromelia pinguin*, *Bromelia plumerii*, *Bauhinia ungulata*, *Callistemon viminalis*, *Centrosema macrocarpum*, *Cordia guanacastensis*, *Malvaviscus arboreus*, *Pithecoctineum crucigerum*, *Stemmedenia obovata*, *Tabebuia ochracea*, and *Vachellia collinsii*. In this model, the total number of “small-patch” FLPVs per individual monkey was the dependent variable, with colour vision phenotype acting as the predictor variable. We treated group membership as a random effect and used the log of the number of observation hours for each group as an offset to control for differences in sampling effort.

Foraging differences due to sex^51^ or possessing a long-wavelength allele^52^ have been predicted for Neotropical monkeys. To control for the possibility that foraging differences were (1) sex-based or (2) resulting from an advantage due to possessing a long-wavelength (561 nm) sensitive allele regardless of whether an individual was a dichromat or trichromat, we tested alternative models for both flower foraging frequency and flower patch visit analyses. First, we tested for sex differences by comparing male and female dichromats of the 561 nm sensitive phenotype, which was the phenotype of most (29/31) of the female dichromats in our study population during the study period. We also ran each model with only females to confirm any effects were not due to colour vision ability differences and weren’t confounded by sex. Secondly, we compared dichromatic males of phenotype 532 to those with the 561 phenotype. We found no significant differences in foraging rates due to sex or from possessing the 561 allele, therefore sex and allele-specific phenotype was not included in the final models (see Supplemental Data S3 for all models with data subsets).

### Data access

Behavioural and spectroscopic data used for this manuscript are provided as Supplemental Data S4.

## Electronic supplementary material

Supplemental Data S1-S3

Manuscript Dataset
