# Supplementary material for: Trichromatic perception of flower colour improves resource detection among New World monkeys
Source: Sci Rep. 2018 Jul 18;8:10883. doi: 10.1038/s41598-018-28997-4 (PMC6052032; doi:10.1038/s41598-018-28997-4)
Supplement: Supplementary file 1 — Supplemental Data S1-S3 [file 41598_2018_28997_MOESM1_ESM.pdf]

# Trichromatic perception of flower colour improves resource detection among New World monkeys

JD Hogan[1], LM Fedigan[1], C Hiramatsu[2], S Kawamura[3] and AD Melin[1*]

1. University of Calgary, Calgary, Canada
2. Kyushu University, Fukuoka, Japan
3. University of Tokyo, Tokyo, Japan

*Corresponding author: amanda.melin@ucalgary.ca

**Supplemental Data S1: Just Noticeable Difference (JND) modelling**

Various "noise" effects influence whether the colour difference between two objects is actually discriminable to an individual. The magnitude of the noise effects on the visual system is determined by both the physical structure of the eye and the physiological limitations of the associated neural pathways. In vertebrate visual systems, the largest contributor of noise is due to the fact that the mid- and long- wavelength sensitive photopigments function as both chromatic and luminance signal receptors, leading to "cross-talk" and signal corruption[1-5]. Estimates of an object's chromaticity to an individual can be achieved by calculating the quantum catch of each photoreceptor present using the following formula[3]:

**Formula 1.** $\mathbf{Q_i = \int_{400}^{700} R(\lambda) I(\lambda) S_i(\lambda) d\lambda}$

This equation calculates the quantum catch (Q) of an object's reflectance R(λ) for photoreceptor "i" within the range of primate vision capabilities (400-700 nm) based on the irradiance spectrum I(λ) of the environment and the spectral sensitivity of the photoreceptor being measured $S_i(\lambda)$. Irradiance data for this study were obtained by ADM in Santa Rosa forest under shaded conditions[6].

**Just Noticeable Difference (JND) Modelling**

To quantify how visible a flower was to each colour vision phenotype, we used Just Noticeable Difference modelling. The minimum chromatic distance at which two objects can be differentiated as discernibly different colours is defined as one just noticeable difference (JND[3]). Objects with increasingly higher JND scores are presumed to be visible under less ideal situations. To approximate a long-distance detection scenario where multiple species would be in the field of view, we calculated a mean chromaticity value for upper and lower leaf surfaces for all species, and then calculated the chromatic distance between a flower part and these mean values. In general, the leaves of all species have similar reflectance properties (i.e. all leaves in this study are green) and our results do not differ if we assess flowers against leaves of each species separately. A flower part was defined as detectable if it was at least 1 JND more visible than leaves for a modelled phenotype, and one phenotype was considered to have a detection advantage over another for a given flower part if the flower was >1 JND more visible

to it. JND scores are determined by the minimum chromatic distance (ΔS) between a target object (flowers in this instance) and its background (leaves) required for differentiation. With only one chromatic pathway, the minimum chromatic distance for dichromats is calculated using:

**Formula 2.** $$\Delta S^2 = \frac{(\Delta f_L - \Delta f_S)^2}{(\Delta \omega_S)^2 + (\Delta \omega_L)^2}$$

Trichromats have three different photopigments, therefore their chromatic distance is calculated using:

**Formula 3.** $$\Delta S^2 = \frac{\omega_S{}^2(\Delta f_L - \Delta f_M)^2 + \omega_M{}^2(\Delta f_L - \Delta f_S)^2 + \omega_L{}^2(\Delta f_S - \Delta f_M)^2}{(\omega_S \omega_M)^2 + (\omega_S \omega_L)^2 + (\omega_M \omega_L)^2}$$

In both equations, $\omega_i$ represents the Weber fractions (which act as the "noise" calculation) associated with human photopigments (S:0.08, M:0.02, L:0.02). The difference between the natural logs of the quantum catches for the target object and the background for a given photopigment (e.g., $\Delta f_L = \ln(Q_{L(FLOWER)}) - \ln(Q_{L(LEAVES)})$) is represented by $\Delta f_L$.

**Supplemental Data S1 Table 1:** Just Noticeable Difference (JND) scores for 21 flower parts of 14 capuchin flower food species. JND values are scored relative to the mean chromatic value of the leaves of 28 plant species common to the Santa Rosa forest. Samples shaded in blue are “small patch” species that produce small crops of monopolizable flowers.

| | | Phenotype Modelled | | | | | |
|---|---|---|---|---|---|---|---|
| **Species** | **Part** | **532/543** | **532/561** | **543/561** | **532** | **543** | **561** |
| *Bauhinia ungulata* | Stamen | 2.40 | 2.65 | 2.59 | 2.41 | 2.39 | 2.27 |
| *Callistemon viminalis* | Petal | 3.57 | 9.71 | 6.39 | 0.80 | 0.65 | 0.85 |
| *Cassia grandis* | Petal | 2.07 | 5.89 | 3.86 | 0.33 | 0.30 | 0.39 |
| *Centrosema macrocarpum* | Bract | 1.76 | 3.23 | 2.53 | 1.58 | 1.49 | 1.26 |
| *Centrosema macrocarpum* | Petal | 5.30 | 13.39 | 10.20 | 4.24 | 3.87 | 2.80 |
| *Centrosema macrocarpum* | Stamen | 3.09 | 3.38 | 3.26 | 3.03 | 3.05 | 2.95 |
| *Cochlospermum vitifolium* | Petal | 6.95 | 9.16 | 7.88 | 6.40 | 6.18 | 7.08 |
| *Curatella americana* | Immature | 2.87 | 3.07 | 3.07 | 2.86 | 2.86 | 2.73 |
| *Diphysa americana* | Petal | 9.04 | 10.79 | 9.81 | 8.62 | 8.87 | 9.28 |
| *Luehea candida* | Bract | 1.48 | 2.70 | 2.16 | 1.20 | 1.16 | 1.03 |
| *Luehea candida* | Immature | 1.33 | 1.83 | 1.64 | 1.32 | 1.29 | 1.17 |
| *Luehea candida* | Petal | 3.11 | 4.00 | 3.56 | 2.92 | 3.00 | 3.10 |
| *Luehea candida* | Stamen | 4.01 | 5.14 | 4.55 | 3.75 | 3.87 | 4.05 |
| *Luehea speciosa* | Bract | 1.12 | 2.02 | 1.61 | 0.93 | 0.95 | 0.97 |
| *Luehea speciosa* | Petal | 3.15 | 3.11 | 3.13 | 3.05 | 3.09 | 3.04 |
| *Malvaviscus arboreus* | Petal | 11.53 | 27.95 | 16.51 | 1.23 | 0.81 | 1.90 |
| *Manilkara chicle* | Immature | 5.62 | 9.69 | 7.25 | 4.06 | 4.79 | 5.36 |
| *Manilkara chicle* | Petal | 1.86 | 3.91 | 2.93 | 1.17 | 1.21 | 1.34 |
| *Stemmedenia obovata* | Petal | 4.77 | 7.23 | 5.84 | 4.07 | 4.27 | 4.63 |
| *Tabebuia ochracea* | Immature | 2.62 | 2.90 | 2.85 | 2.61 | 2.61 | 2.47 |
| *Vachellia collinsii* | Stamen | 3.60 | 7.83 | 5.44 | 2.17 | 2.47 | 2.99 |

**Supplemental Data S2: Support Vector Machine (SVM) modelling**

As a complimentary approach to JND modeling, we use Support Vector Machine (SVM) modelling, a supervised machine learning classification algorithm[7], to predict for which flower species trichromats may have an advantage over dichromats in long distance detection against leafy backgrounds. Once quantum catch for each photoreceptor present in a phenotype was known (obtained during Just Noticeable Difference modelling, see Supplemental Data S1), we calculated how an object stimulated the lightness (luminance) vision pathway ($Q_L$+$Q_M$), the blue-yellow pathway ($Q_S/(Q_L+Q_M)$), and, for trichromats, the red-green pathway ($Q_L/(Q_L+Q_M)$). In our models the spectral sensitivity functions of the three cones were obtained by normalizing the functions for corneal sensitivity. The quantum catch of the three receptors under a given illumination were all set to be 1 for the light emitted from a hypothetical white surface which reflects 100% of the illumination light. This normalization was carried out based on the assumptions of color constancy, under which the monkeys' eyes would adapt to the varying spectral compositions of illumination so that this surface would appear white[8].

These quantum catch values are then used with the LIBSVM[9] extension of MATLAB (v.2014b). The SVM creates a hyperplane using "leave one out" methodology. The hyperplane created maximizes the chromatic distance between leaf and flower data points using the chromaticity values for all samples (leaf and flower parts) except for one, which it then attempts to classify as a flower or a leaf based off the generated hyperplane. We repeated this for every plant part, for every colour vision phenotype, and determined how successful a given phenotype was at classifying flowers correctly. Because dichromats may use achromatic visual

pathways to improve foraging efficiency[6], we ran each model separately with lightness included to determine if this improved dichromat success.

All Support Vector Machine (SVM) models using trichromat phenotypes were able to correctly categorize a majority of flowers, whereas most flowers were incorrectly categorized in dichromatic models (S2 Figure 1, S2 Table 1). Adding lightness to the SVM models increased the success of dichromats and slightly decreased trichromatic success. The majority of flower parts (12/21) had luminance values that overlapped with sampled leaves (S2 Figures 2 and 3).

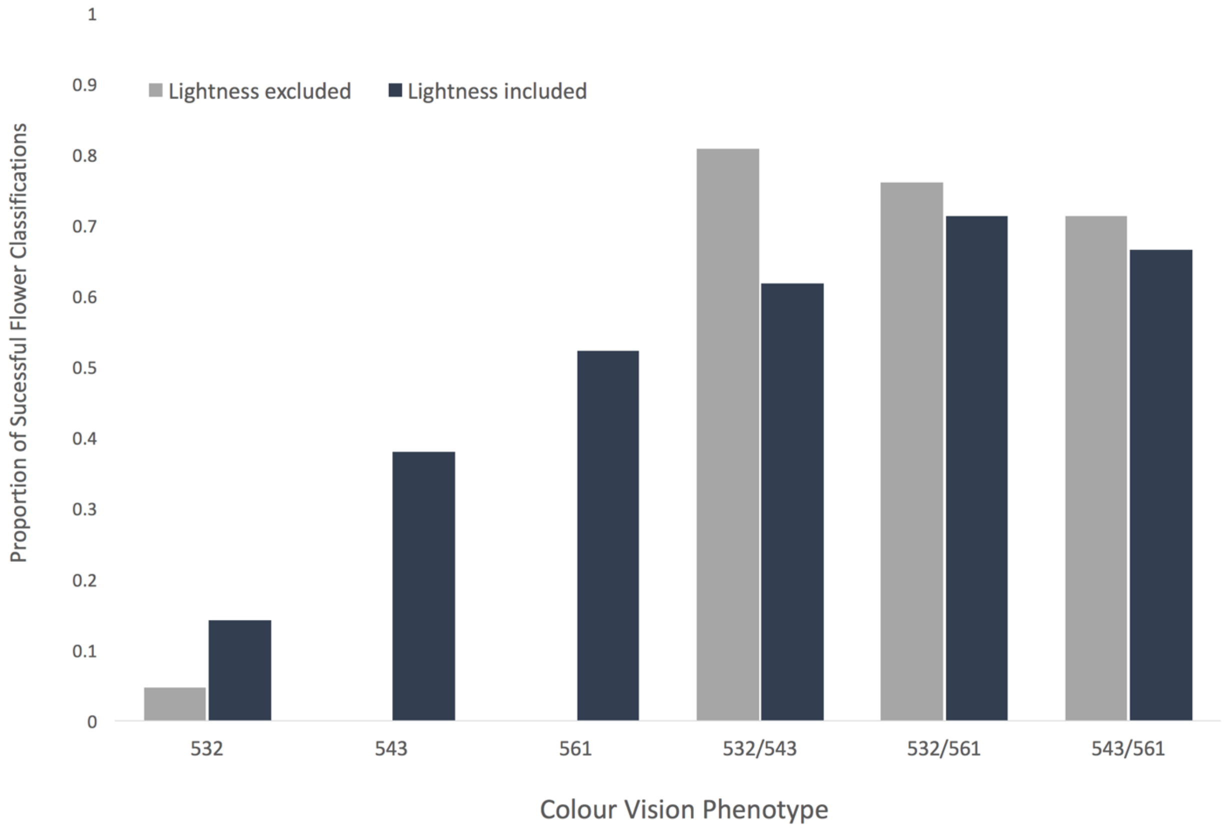


**S2 Figure 1.** Results from support vector machine (SVM) modelling for each capuchin colour vision phenotype. Trichromats outperformed dichromats with or without lightness included, although dichromat success improved with lightness cues available while trichromats performed marginally poorer.

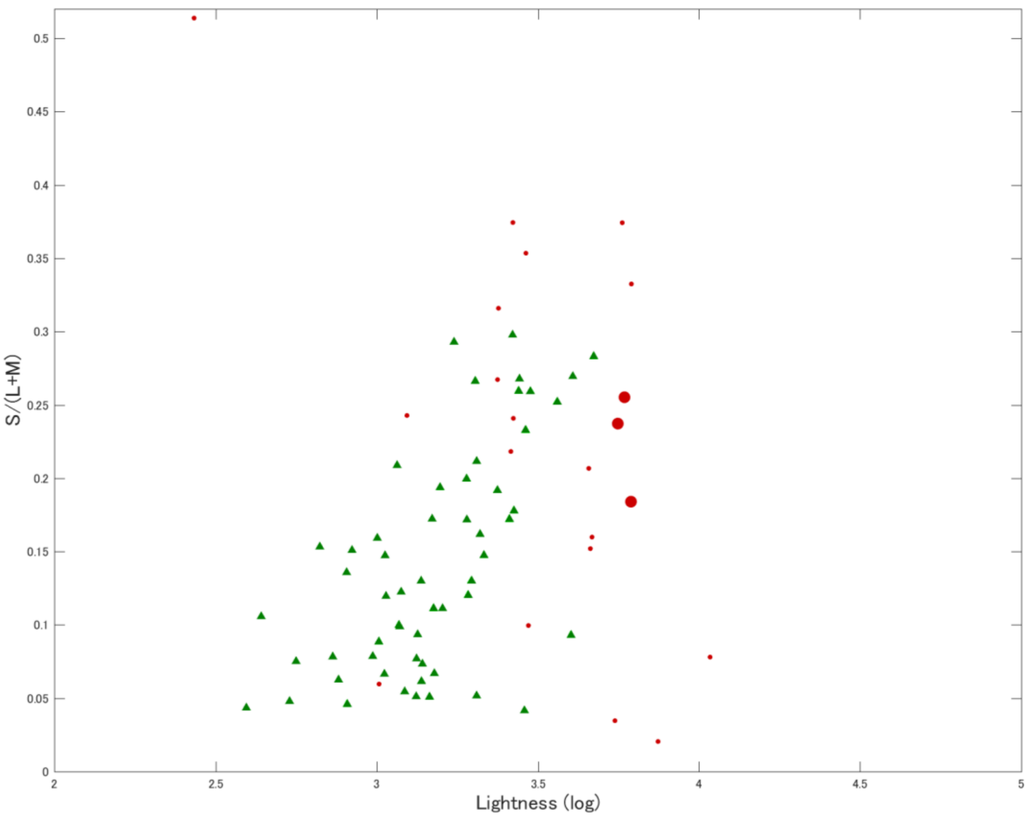


**S2 Figure 2.** Support Vector Machine (SVM) results for dichromat phenotype 532, with luminance included in the model. Green triangles represent the positions of leaves in the colour space, large red circles indicate the colour space of flowers that were correctly identified (3/21) and small red circles indicate the colour space of flowers the SVM incorrectly identified as leaves (18/21) for this phenotype. The y-axis of this figure represents the range of variation within the blue-yellow colour space for this phenotype, while the x-axis represents the lightness properties of the object. Lightness values for the majority (12/21) of flowers fell within the range of leaves, suggesting this cue is not particularly helpful for detecting most flower species. The red-green colour vision pathway is not available to dichromats, and thus it can not aid in flower identification.

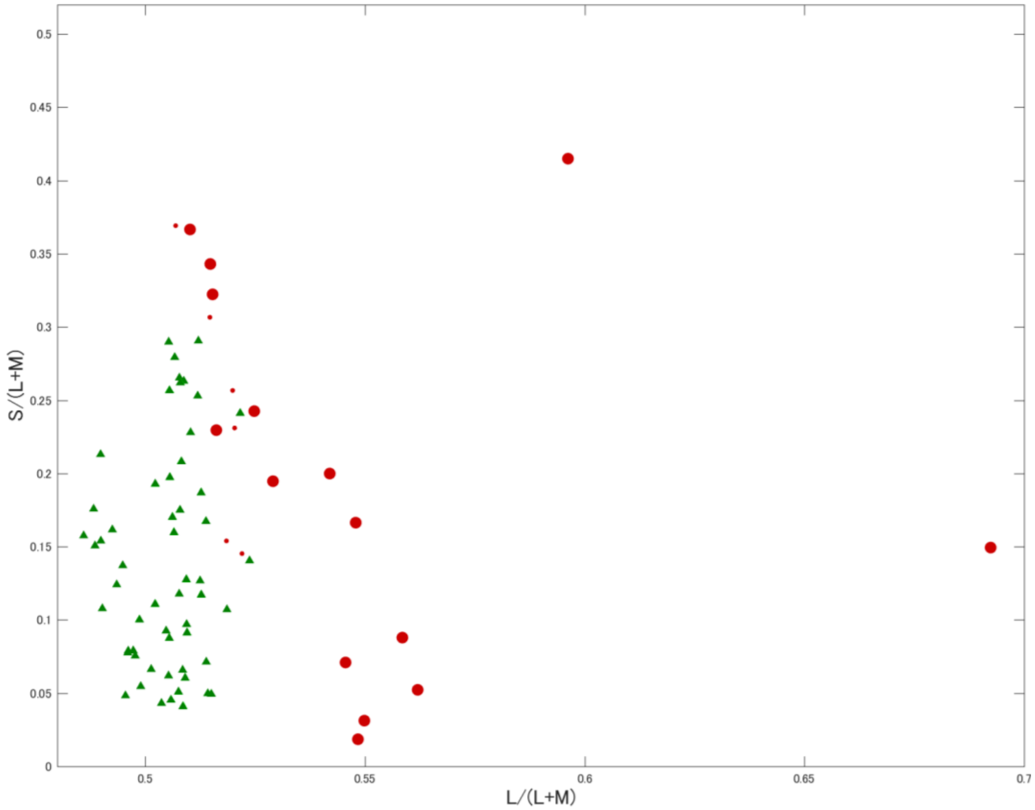


**S2 Figure 3.** Support Vector Machine (SVM) results for trichromat phenotype 532/561, with luminance included in the model (not visualized). Green triangles represent the positions of leaves in the colour space, large red circles indicate the colour space of flowers that were correctly identified (15/21) and small red circles indicate the colour space of flowers the SVM incorrectly identified as leaves for this phenotype (6/21). The y-axis of this figure represents the range variation within the blue-yellow colour vision pathway for this phenotype, while the x-axis represents the red-green range. Although lightness adds a third dimension to the colour space from which flowers can vary from background leaves (not included in this figure), the lightness values for the majority (12/21) of flowers fell within the range of leaves, suggesting this cue is not particularly helpful for detecting most flower species. With luminance excluded from SVM modelling, this trichromat phenotype correctly identified one extra flower (16/21).

**S2 Table 1.** The success of Support Vector Machine (SVM) analysis at categorizing a given flower part for each color vision phenotype, when lightness is included (white columns) and excluded (shaded columns). "Middles" is a category used to denote the middle, non-petal structures of a flower, typically this was the reproductive organs. "Small patch" species are shaded in blue.

| | **Peak Spectral Sensitivity** | | | | | | | | | | | |
|---|---|---|---|---|---|---|---|---|---|---|---|---|
| | 532 | 532 | 543 | 543 | 561 | 561 | 532/ 543 | 532/ 543 | 532/ 561 | 532/ 561 | 543/ 561 | 543/ 561 |
| **Lightness Included** | No | Yes | No | Yes | No | Yes | No | Yes | No | Yes | No | Yes |
| **Flower Species and Part** | | | | | | | | | | | | |
| *Bauhinia ungulata*- Stamen | - | - | - | - | - | - | + | - | + | - | + | - |
| *Callistemon viminalis*- Petal | - | - | - | - | - | - | + | + | + | + | + | + |
| *Cassia grandis*- Petal | - | + | - | + | - | + | + | + | + | + | + | + |
| *Centrosema macrocarpum*- Bract | - | + | - | + | - | + | + | + | + | + | + | + |
| *Centrosema macrocarpum*- Petal | + | + | - | - | - | - | + | + | + | + | + | + |
| *Centrosema macrocarpum*- Stamen | - | - | - | + | - | + | + | + | + | + | - | + |
| *Cochlospermum vitifolium*- Petal | - | - | - | - | - | + | + | + | + | + | + | + |
| *Curatella americana*- Immature | - | - | - | - | - | - | + | - | + | + | + | + |
| *Diphysa americana*- Petal | - | - | - | + | - | + | + | + | + | + | + | + |
| *Luehea candida*- Bract | - | - | - | - | - | - | + | - | + | - | + | - |
| *Luehea candida*- Immature | - | + | - | + | - | + | - | - | - | + | - | - |
| *Luehea candida*- Petal | - | - | - | + | - | + | - | - | - | - | - | - |
| *Luehea candida*- Stamen | - | - | - | - | - | - | - | - | - | - | - | - |
| *Luehea speciosa*- Bract | - | - | - | - | - | + | - | - | - | - | - | - |
| *Luehea speciosa*- Petal | - | - | - | - | - | - | + | - | - | - | - | - |
| *Malvaviscus arboreus*- Petal | - | - | - | - | - | - | + | + | + | + | + | + |
| *Manilkara chicle*- Immature | - | - | - | - | - | - | + | + | + | + | + | + |
| *Manilkara chicle*- Petal | - | - | - | - | - | + | + | + | + | + | + | + |
| *Stemmedenia obovata*- Petal | - | - | - | + | - | + | + | + | + | + | + | + |
| *Tabebuia ochracea*- Immature | - | - | - | + | - | + | + | + | + | + | + | + |
| *Vachellia collinsii*- Stamen | - | - | - | - | - | - | + | + | + | + | + | + |
| **TOTAL CORRECT** | **1** | **3** | **0** | **8** | **0** | **11** | **17** | **13** | **16** | **15** | **15** | **14** |
| **PERCENTAGE CORRECT** | **5%** | **14%** | **0%** | **38%** | **0%** | **52%** | **81%** | **62%** | **76%** | **71%** | **71%** | **67%** |

**Supplemental Data S3: Behavioural data subsets results**

**S3 Table 1.** Generalized linear mixed modelling (GLMM) results for data subsets using the Small Patch Visit (SPV) dataset. Female trichromats were found in small patch visits significantly more frequently than female dichromats were, but no significant differences were detected between male and female individuals of the dichromat 561 phenotype, nor between males of the 532 and 561 dichromat phenotypes.

| Model Description | Estimate | SE | z | p |
|---|---|---|---|---|
| Females: trichromatic vs dichromatic | 0.9458 | 0.3614 | 2.618 | **0.00886** |
| Dichromat 561 phenotype: males vs females | 0.3569 | 0.6160 | 0.579 | 0.562 |
| Males: Dichromat 532 vs 561 phenotype | -0.8019 | 0.7150 | 1.121 | 0.262 |

**S3 Table 2.** Generalized linear mixed modelling (GLMM) results for data subsets using both scan sampling flower foraging frequency dataset. No significant differences were detected between female trichromats and dichromats, male and female individuals of the dichromat 561 phenotype, nor between males of the 532 and 561 dichromat phenotypes.

| Model Description | Estimate | SE | z | p |
|---|---|---|---|---|
| Females: trichromatic vs dichromatic | 0.2887 | 0.2683 | 1.076 | 0.282 |
| Dichromat 561: males vs females | 0.3663 | 0.4225 | 0.867 | 0.386 |
| Males: Dichromat 532 vs 561 phenotype | -0.7661 | 0.5453 | 1.405 | 0.16 |

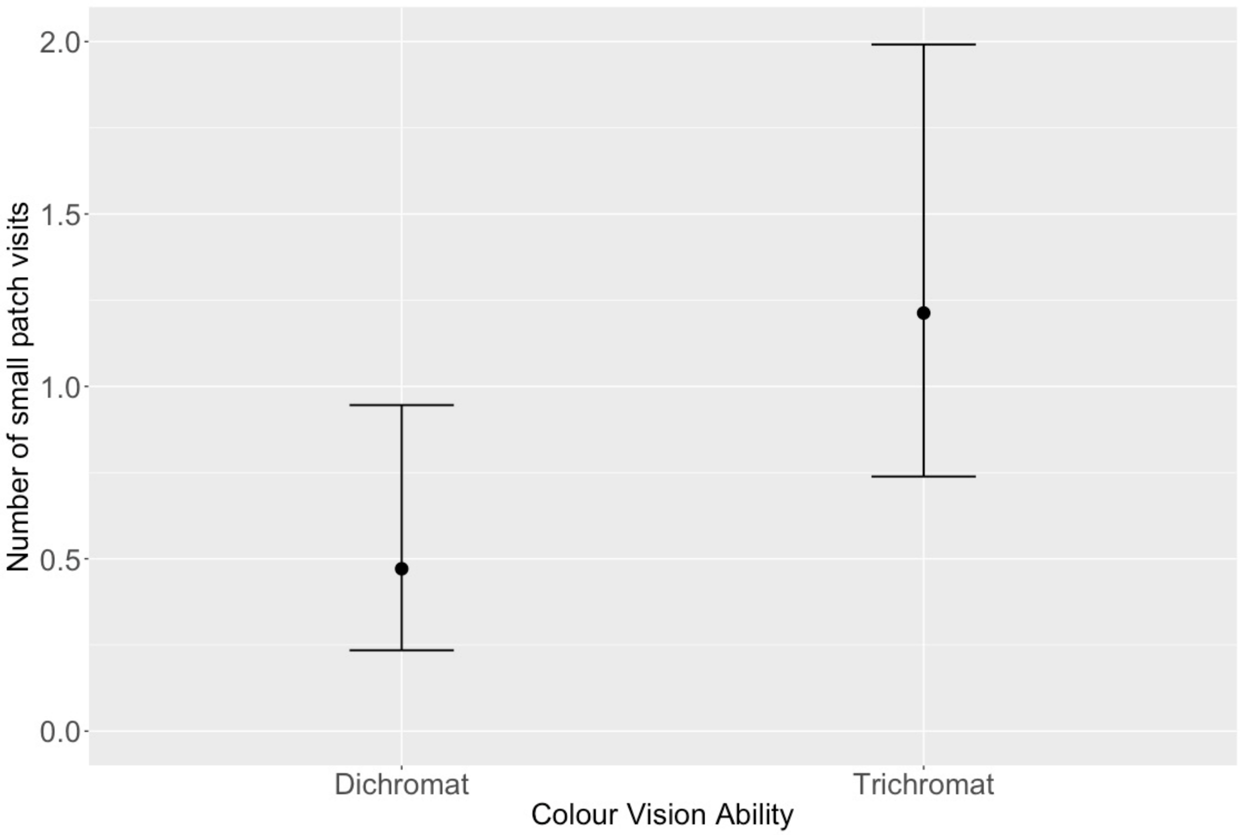


**S3 Figure 1.** Least square means estimation of the number of visits to small patch flowers by female dichromats and trichromats. Trichromats visited small patches significantly more frequently ($p = 0.00886$).

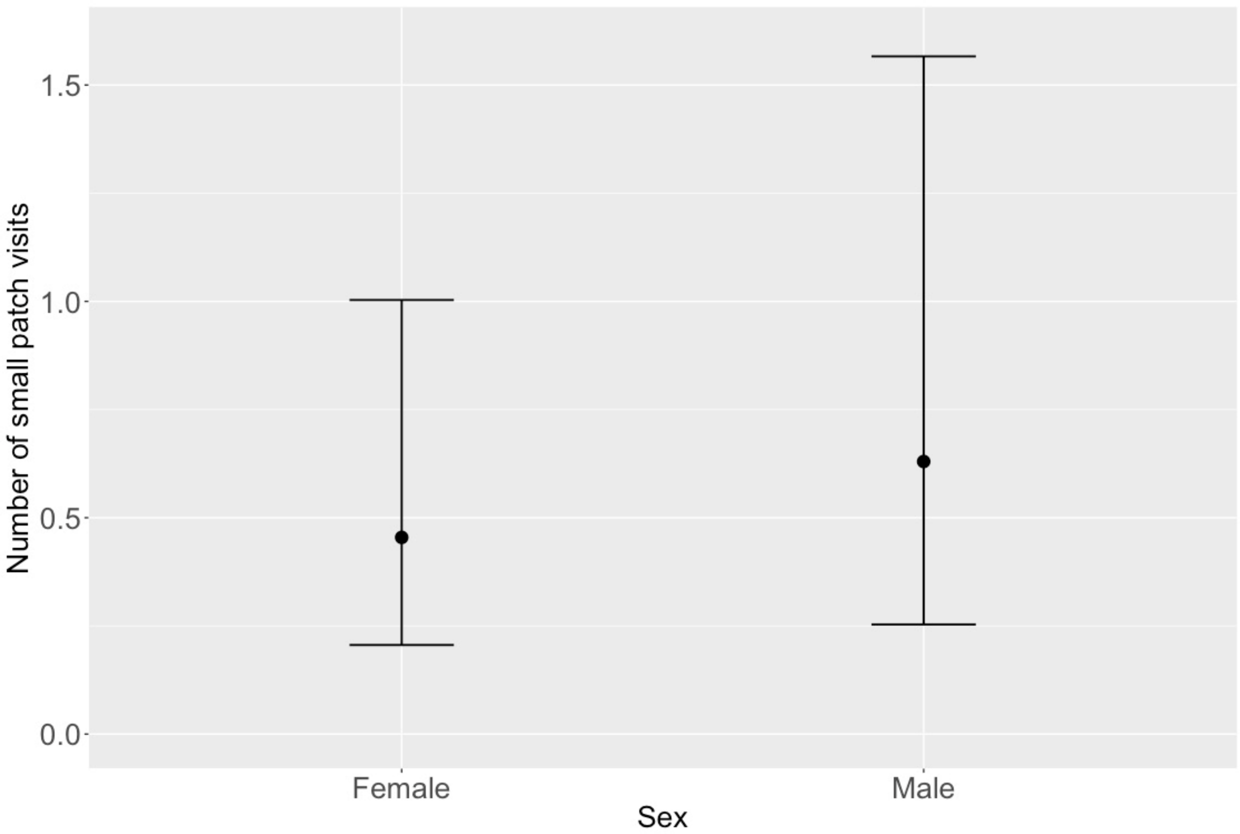


**S3 Figure 2**. Least square means estimation of the number of visits to small patch flowers by females with the Dichromat 561 phenotype vs males of the same phenotype reveal no significant difference.

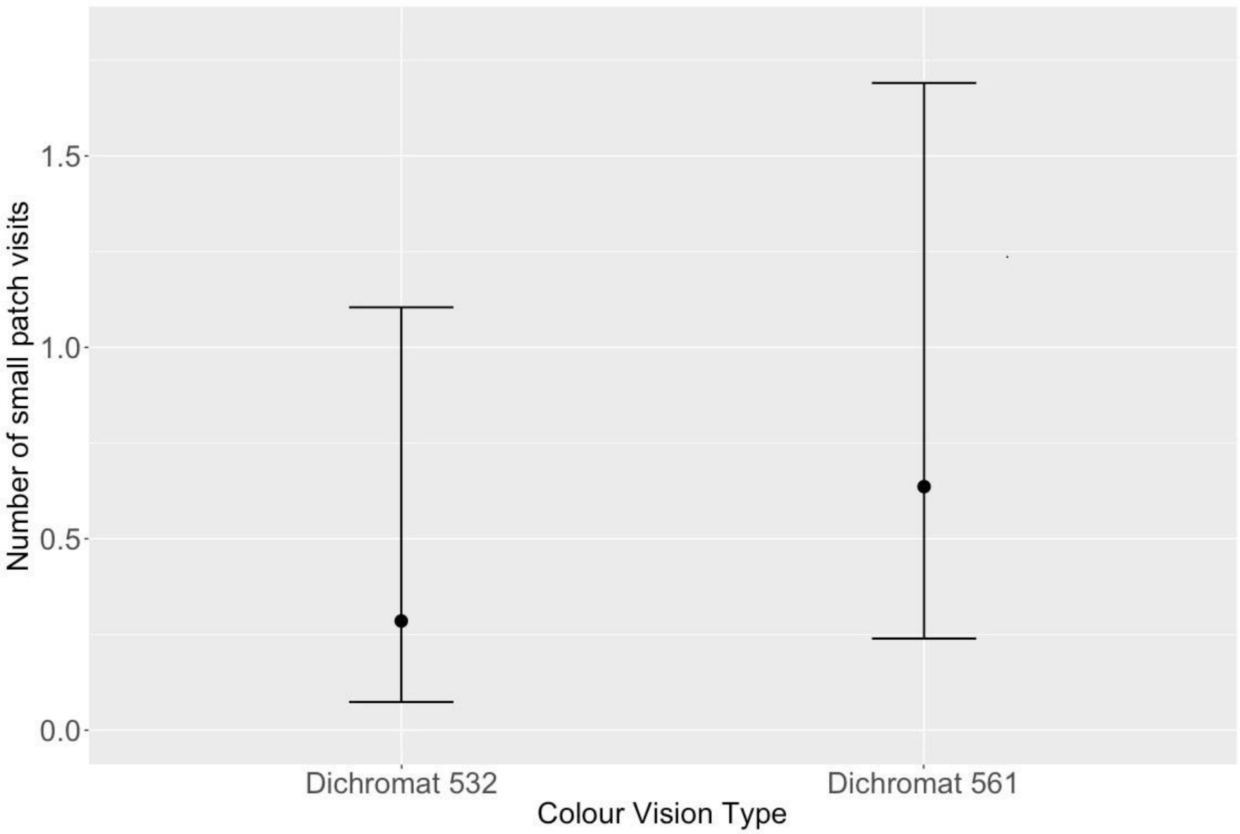


**S3 Figure 3**. Least square means estimation of the number of visits to small patch flowers by males with Dichromat 532 phenotype vs the Dichromat 561 phenotype. There was no significant difference in patch visits between these phenotypes.

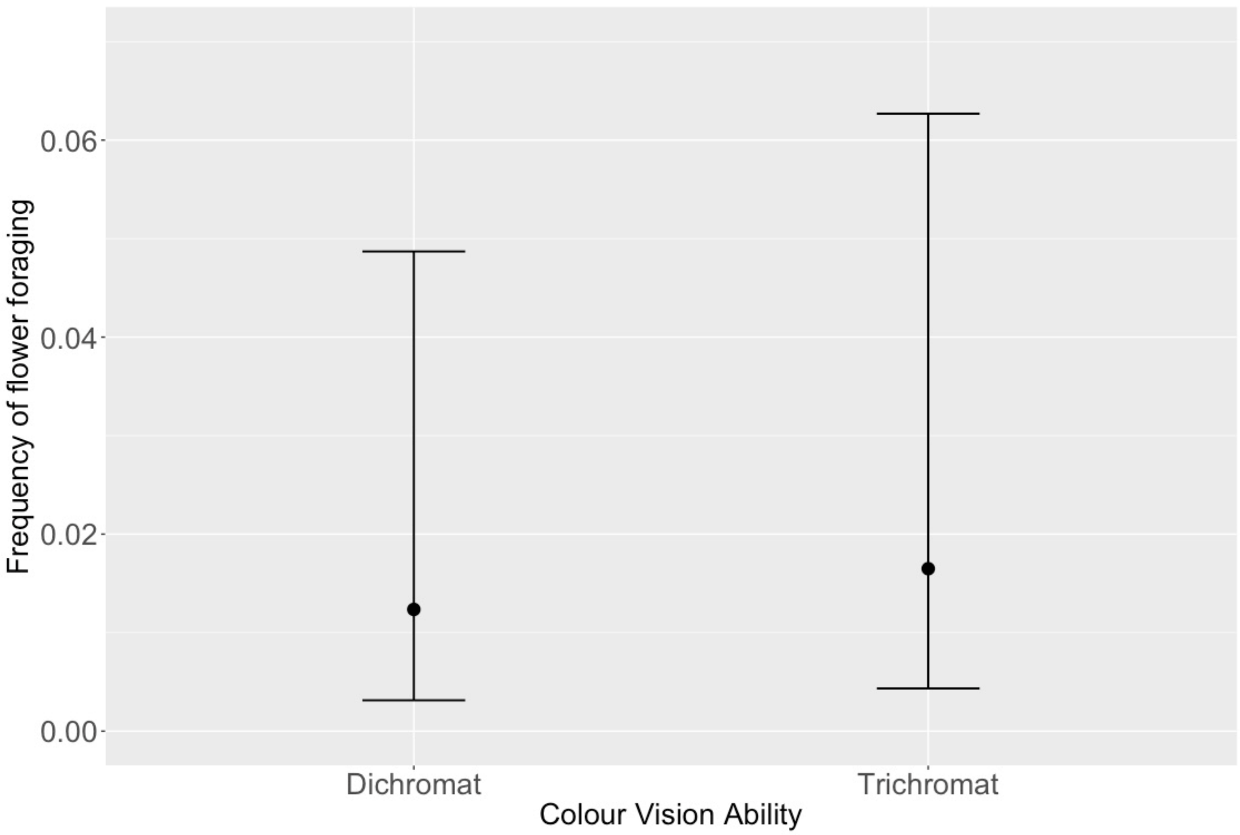


**S3 Figure 4.** Least square means estimation of the frequency of flower foraging during scan sampling by female dichromats and trichromats. There is no significant difference in the frequency of flower foraging between individuals with different colour vision abilities.

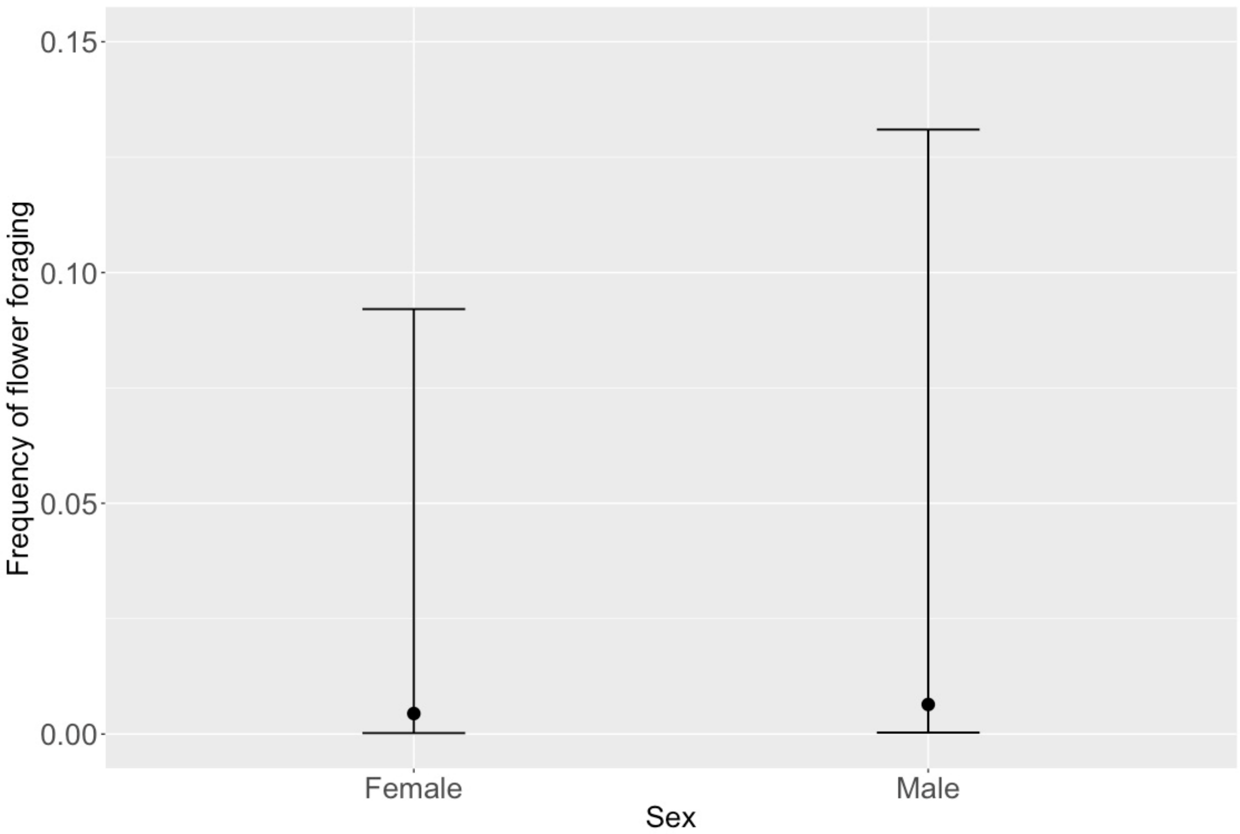


**S3 Figure 5.** Least square means estimation of the frequency of flower foraging during scan sampling by females with the Dichromat 561 phenotype vs males of the same phenotype. There is no significant difference in the frequency of flower foraging between sexes for this colour vision phenotype.

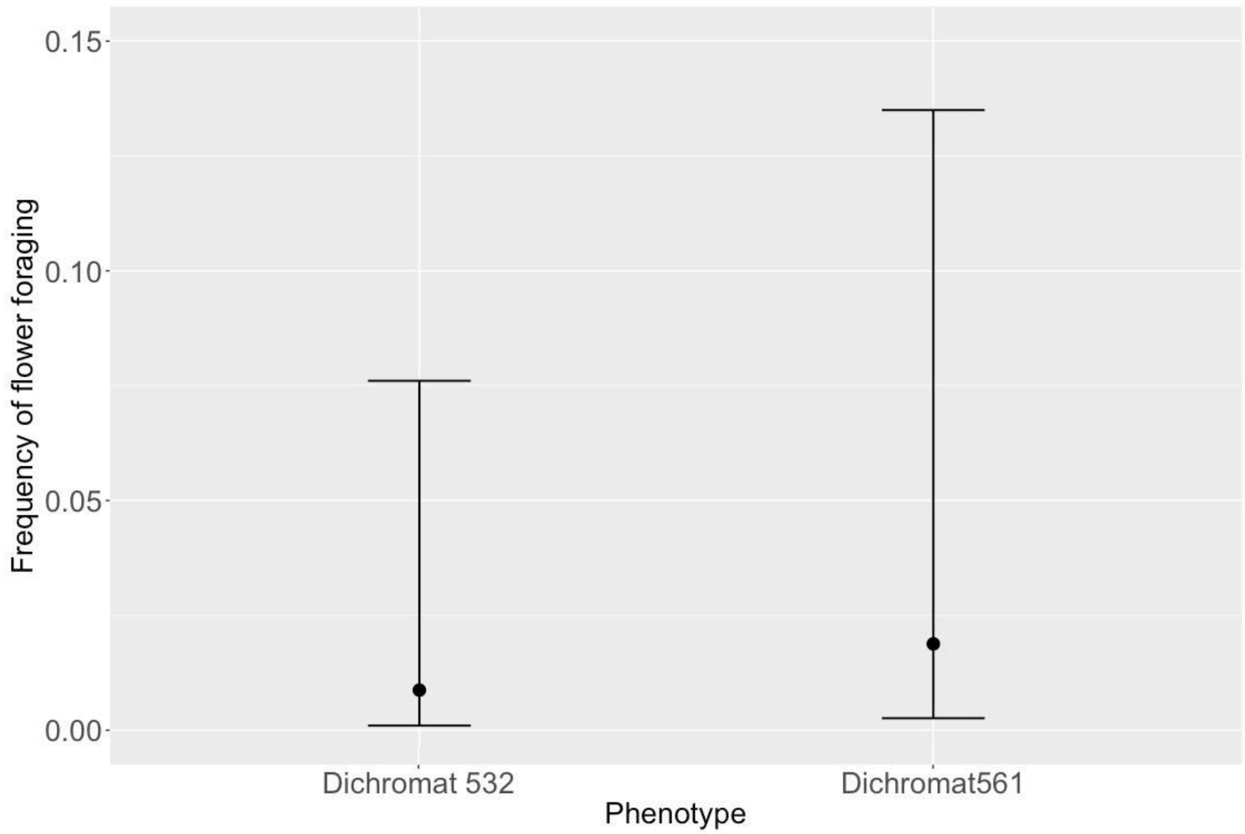


**S3 Figure 6.** Least square means estimation of the frequency of flower foraging during scan sampling by males of the Dichromat 532 phenotype and males of the Dichromat 561 colour vision phenotype. There is no significant difference in the frequency of flower foraging between individuals of different phenotypes.

# SUPPLEMENTAL DATA REFERENCES

1. Osorio, D. & Vorobyev, M. Photoreceptor spectral sensitivities in terrestrial animals: adaptations for luminance and colour vision. Proc. R. Soc. B Biol. Sci. **272**, 1745–1752 (2005).

2. Kelber, A., Vorobyev, M. & Osorio, D. Animal colour vision–behavioural tests and physiological concepts. Biol. Rev. **78**, 81–118 (2003).

3. Osorio, D. & Vorobyev, M. Colour vision as an adaptation to frugivory in primates. Proc. R. Soc. Lond. B Biol. Sci. **263**, 593–599 (1996).

4. Govardovskii, V. I., Fyhrquist, N., Reuter, T. O. M., Kuzmin, D. G. & Donner, K. In search of the visual pigment template. Vis. Neurosci. **17**, 509–528 (2000).

5. Nagle, M. G. & Osorio, D. The tuning of human photopigments may minimize red-green chromatic signals in natural conditions. Proc. R. Soc. Lond. B Biol. Sci. **252**, 209–213 (1993).

6. Hiramatsu, C. *et al.* Importance of Achromatic Contrast in Short-Range Fruit Foraging of Primates. *PLoS ONE* **3,** e3356 (2008).

7. Cortes, C. & Vapnik, V. Support-vector networks. *Mach. Learn.* **20,** 273-297 (1995).

8. Hiramatsu, C. *et al.* Color-vision polymorphism in wild capuchins (*Cebus capucinus*) and spider monkeys (*Ateles geoffroyi*) in Costa Rica. *Am. J. Primatol.* **67,** 447–461 (2005).

9. Chang, C. C. & Lin, C. J. LIBSVM: a library for support vector machines. *ACM Trans. Intell. Syst. Technol. TIST* **2,** 27 (2013).
